# Improving vehicle tracking rate and speed estimation in dusty and snowy weather conditions with a vibrating camera

**DOI:** 10.1371/journal.pone.0189145

**Published:** 2017-12-20

**Authors:** Nastaran Yaghoobi Ershadi

**Affiliations:** E.T.S. de Ingenieros de Telecomunicación, Universidad Politécnica de Madrid, Madrid, Spain; Chongqing University, CHINA

## Abstract

Traffic surveillance systems are interesting to many researchers to improve the traffic control and reduce the risk caused by accidents. In this area, many published works are only concerned about vehicle detection in normal conditions. The camera may vibrate due to wind or bridge movement. Detection and tracking of vehicles is a very difficult task when we have bad weather conditions in winter (snowy, rainy, windy, etc.), dusty weather in arid and semi-arid regions, at night, etc. Also, it is very important to consider speed of vehicles in the complicated weather condition. In this paper, we improved our method to track and count vehicles in dusty weather with vibrating camera. For this purpose, we used a background subtraction based strategy mixed with an extra processing to segment vehicles. In this paper, the extra processing included the analysis of the headlight size, location, and area. In our work, tracking was done between consecutive frames via a generalized particle filter to detect the vehicle and pair the headlights using the connected component analysis. So, vehicle counting was performed based on the pairing result, with Centroid of each blob we calculated distance between two frames by simple formula and hence dividing it by the time between two frames obtained from the video. Our proposed method was tested on several video surveillance records in different conditions such as dusty or foggy weather, vibrating camera, and in roads with medium-level traffic volumes. The results showed that the new proposed method performed better than our previously published method and other methods, including the Kalman filter or Gaussian model, in different traffic conditions.

## 1. Introduction and previous works

Today, many researchers are attracted to traffic because it has become a significant problem in our life. Traffic contains useful information such as site selection and engineering which can be used for different purposes. Due to rapid developments in multimedia and wireless communication, video plays a main role in traffic management. In traffic engineering, the detection and tracking of vehicles potentially allow for traffic flow analysis, incident detection and reporting, and automated solutions to queue management. Different vehicle monitoring systems have been developed that use the video in intelligent traffic systems. Besides different advantages of the video-based method, a huge digital processing power to extract the necessary information from the image data [1, 2] can be considered as the main disadvantage of such systems. Counting is a basic function used in many different approaches to detect and count the number of vehicles [3]. Most of the methods in this area have focused on two challenging parts of moving vehicle segmentation and recount cancellation strategy in different conditions such as high occlusion, cluttered background, dusty weather, and vibrating camera. A vehicle detection approach was presented by Cao and his colleagues [3] in which vehicles are detected in static images using color and edge. In their work, the vehicles were first extracted from the background only based on color and corner, edge maps, and wavelet coefficient by a cascade multi-channel classifier to verify possible candidates. Another automatic vehicle system classification was presented by Ilyas et al. [1] that operates based on pixel wise relations in a region. They used edge detection to calculate local features of the image, and color conversion to segment the vehicle. Also, they used a Dynamic Bayesian Network for their classification. Vehicle detection based on high vertical symmetry was developed by Litzenberger et al. [2] to facilitate vehicle detection on a video stream. Also, Sun and his colleagues [4] proposed a new method to detect vehicles based on feature extraction and classification process. They used the Gabor filter to extract features and used support vector machine for classification. Meshram and his colleague in [5] first construct initial background image according to the real-time situation of traffic environment and then segmented the current frame into foreground region and background region accurately using the combined method of inter frame difference and subtraction method. Their method can detect moving vehicles fast and accurately in complex traffic situation. Also, [6] used connected component labeling technique for complex conditions.

Since reliable vehicle detection and tracking is a critical problem for most of the existing methods, we decided to present a new system to count vehicles even in dusty weather, vibrated camera, occlusion, and background noise. These conditions are hard to deal with, and not many works have been published work in such situation. Zhang et al. [7] proposed a video-based vehicle detection and classification system for vehicle counting, operating under 3 different conditions: normal weather, heavy shadow in the images, and light rain with slight camera vibration. Even though their results are quite promising, their system cannot handle longitudinal vehicle occlusions, severe camera vibrations and head light reflection problems.

Ikoma et al. [8] proposed a method for car tracking based on bicycle specific motions in vertical vibration and angular variation via prediction and likelihood models, and using particle filter for state estimation. The method was tested only under normal weather condition observing that the estimation was limited as the lighting conditions were reduced. Finally, Afolabi et al. [9] used monocular cameras, mounted on moving vehicles such as quadcopters or similar unmanned aerial vehicles (UAVs). These cameras are subjected to vibration due to the constant movement experienced by these vehicles and consequently, the captured images are often distorted. Their approach used the Hough transform for ground line detection under normal weather conditions and concentrate on reducing the effect of the camera vibration. Our approach outperforms this point thanks to the implementation of our generalized particle filter. Yaghoobi Ershadi et al. [10] implemented a method by particle filter. The results showed that proposed method was robust in complex conditions such as bright nights, snowy and dusty weather with vibrated camera (due to the wide range of traffic condition and varying speeds in different lanes). However, some improvements may still be included, as the detection rate may still be enhanced. We have detected that white cars are hardly detected under snowing conditions far from the vibrated camera. Additionally, the background subtraction method may be affected by vibrations of the camera or background movement due to windy conditions. Due to that, we plan to enhance our system by extending the particle filter functionality.

In our proposed system, the vehicles entering the scene are detected and tracked throughout the video. The input video is first fed to the algorithm; then, background subtraction is performed based on the video frames. The proposed algorithm uses this step to distinguish the vehicles from the surroundings. In the next stage, a neighborhood analysis is used to group some detected parts in the scene. After that, to identify small changes on the monitored road filtering and tracking is performed using a generalized particle filter until the vehicles leave the scene. For calculating the distance between vehicles we used centroid of each blob between two frames then dividing it by time between two frames obtained from the video.

The remainder of this paper is organized as follows. In the next section, we will describe our proposed method and different parts of our system. Background subtraction in section III. In section IV we have new tracking method, Experimental result in section V. Finally, some concluding remarks are made in SectionVI.

## 2. System overview

**Fig 1:**
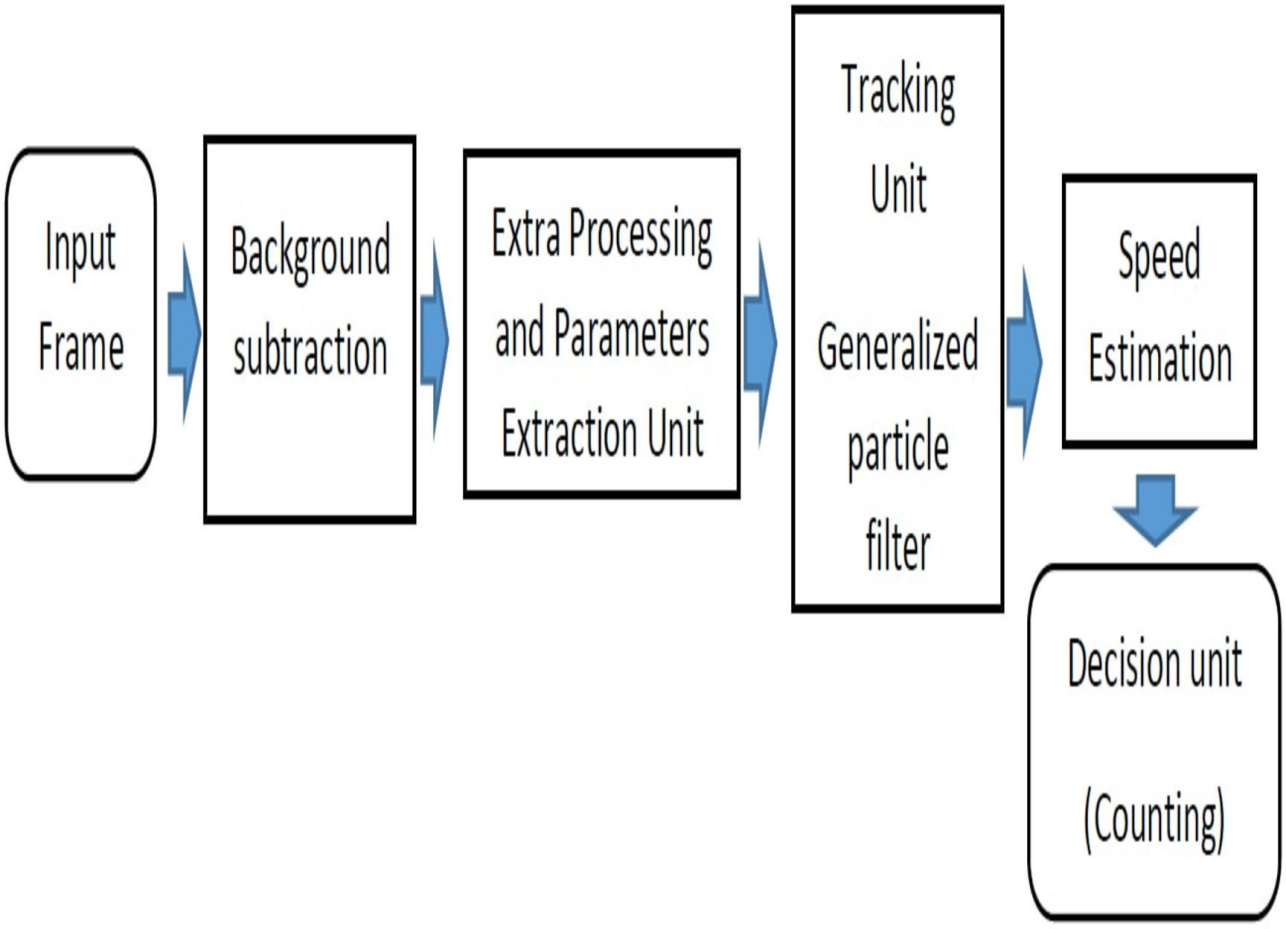
Flowchart of proposed algorithm.

As depicted in Fig 1, our new proposed system comprises four main components: a standard background subtraction algorithm [1] which is mixed with an extra processing module, A generalized particle filter based tracker, Speed estimation and a decision making block. All these four parts are combined into a tightly coupled framework. For each input frame, the background subtraction algorithm segments the areas, and each of them is assumed to be either a vehicle or a group of vehicles. Also, since we considered videos recorded in unfavorable conditions such as dusty weather and vibrated camera, we used an extra processing unit to enhance the accuracy of our vehicle segmentation algorithm and provide better parameters for our tracking unit. For this purpose, we analyzed the headlight size, location, and area for regions resulting from background subtraction algorithm. After that, we used a generalized particle filter to track and find each vehicle in the next frame then calculating speed estimation. Our tracker maintained the trajectory of each vehicle over time whose parameters were sent to the decision-making module. Finally, these trajectories were used in the vehicle counting applications. In the following, we will describe these four parts of our algorithm in more detail.

## 3. Background subtraction and extra processing

Our proposed method can be used even if the camera is vibrated; hence, the background seen in the camera images is nearly dynamic. Since the next stages of our algorithm could be affected by the result of this section, a robust and accurate algorithm was desired to perform our background subtraction section. Our approach computed a model for the background and updated it continuously over the video scenes. For this section, we used the algorithm in [11] due to its robustness and high detection performance. The background of the videos is assumed to be static or of little movement at least between consecutive frames due to the small time interval between them. This approach first produced a model based on the statistical histogram of the background for each pixel and collected the color co-occurrences to increase the accuracy of the dynamic background and classified the background. To adapt the model more, the algorithm updated the model in each frame using a weighted average filter. For this purpose, we calculate the average value and standard deviation ratio in each frame and update the previous values based on a forget factor. Also, in this paper, extra processing was used to tackle the problem of hollow phenomenon. For this purpose, the headlight size, location, and area were analyzed, and morphological operations were used. Thinning is a morphological operation. It used to remove selected foreground pixels from binary images. The thinning here is an operation related to the hit-and-miss transform. The thinning of an image *I* by a structuring element *J* is thin (I,J) = I- hit-and-miss (I,J). The subtraction is a logical subtraction defined by X-Y = X ∩ NOT Y. In this case, if the foreground and background pixels in the structuring element exactly match foreground and background pixels in our image, then the image pixel underneath the origin of the structuring element is set to background (zero). Otherwise it is left unchanged. Note that, in this paper the structuring element must always have a one or a blank at its origin if it is to have any effect.

Since we face different blobs in the results obtained from the background subtraction step and not all of them are related to vehicles and may result from the dusty weather with a vibrated camera, extra processing plays a key role in our final results. Therefore, relevant pixels were grouped and the headlight size, location, and area of blobs were analyzed to remove irrelevant blobs and reduce false detections.

Once the Vehicle is detected using the above steps then next we will calculate the centroid of the detected vehicle and draw the bounding box by regionprops function of the MATLAB.

**Fig 2:**
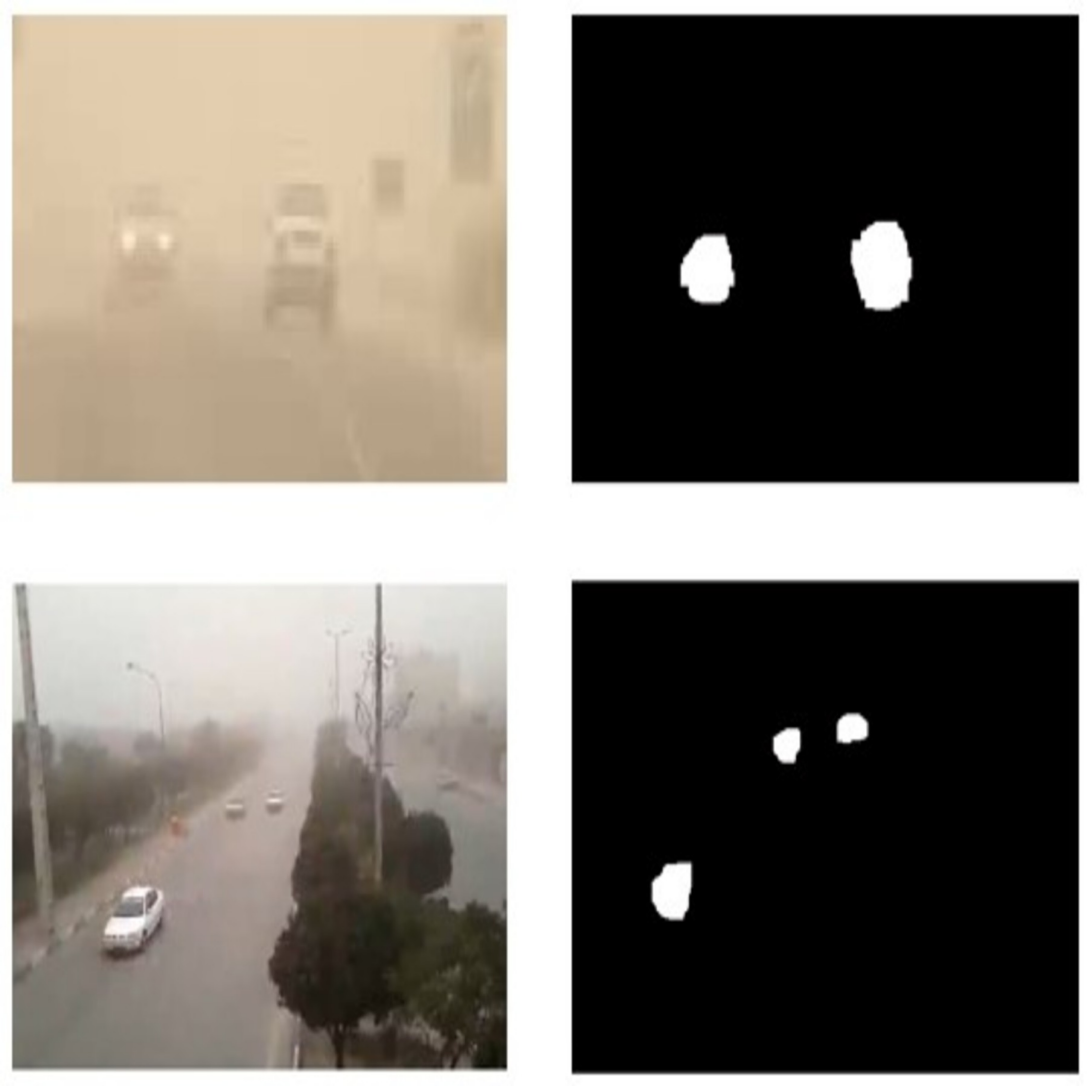
Result of background subtraction algorithm.

The sample results from background subtraction in different weather condition are illustrated in Fig 2.

### 3.1 Counting the number of vehicles

In order to count the number of vehicles in each frame, we needed to verify the vehicles based on their information in the last frames. For this purpose, we used a generalized particle filter and initialized its particles with a group of data corresponding to each vehicle and verified at the last stage. Each group of data was extracted from the region within the bounding box of each verified blob in the last stage and based on the value of the pixels. One obvious result of connected components analysis is that the objects in an image can be readily counted. A connected component analysis beside our tracking procedure could be useful to refine our search in the current frame.

Vehicle counting uses the information of the rectangular box that encloses the detected vehicle described in section 3.

**Fig 3:**
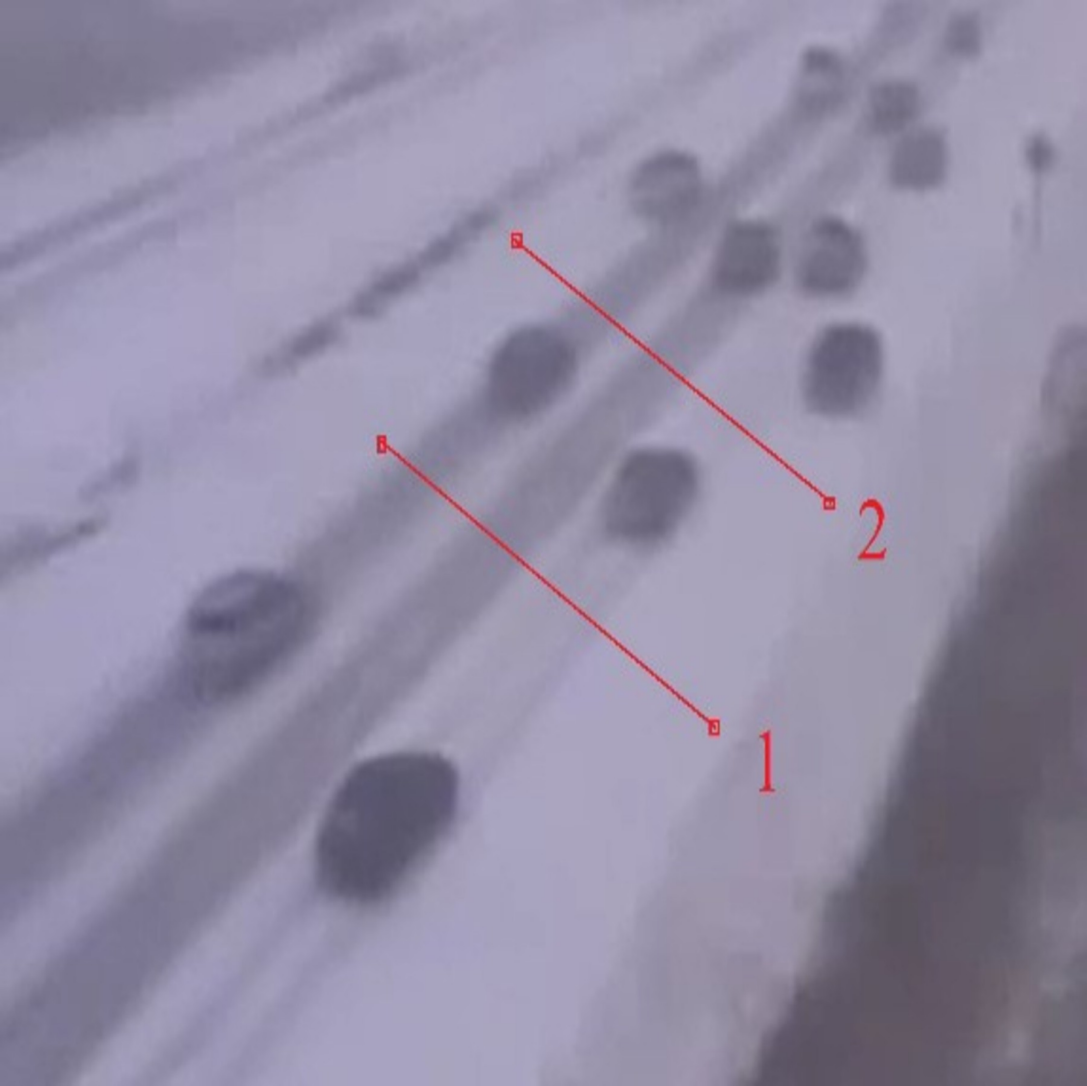
Example of regions of interest on the highway with low light.

We defined a region of interest of the highway where the vehicles are going to be counted. This region is previously defined by the user. Fig 3 shows an example of regions of interest on the highway in low light with snowy weather conditions. Line 1 detects the entry of vehicles into the regions of interest. When a rectangle touches line 1, the vehicle is counted. Line 2 in the figure indicates the end of the regions of interest.

## 4. Tracking by generalized particle filter

Vehicle tracking is a powerful tool in traffic monitoring. Tracking allow us to have: 1) intelligently combine the independent blobs, that move close to each other and almost rigidly, to realize that they are part of the same vehicle; 2) help solving possible problems of occlusions due to the perspective of the camera; and 3) determine an estimation of the speed of the vehicle, once the information of the frame rate of the camera is known.

Many works have already been published in the field of traffic monitoring, making use of recursive estate estimate filters, such as the Kalman filter. This filter works well under the assumption of Gaussian distributed state variables, and linear state equations. However, these conditions are not fulfilled when working with vibrated cameras, in which the dynamics are almost random, and the state equations are difficult to model. This limitation can be overcome through the use of the particle filter, which does not assume any specific movement, and provides good flexibility to obtain a good tracking between frames (as long as the selected features are properly selected).

To the best of our knowledge, there is no previous work using Kalman filtering and vibrated cameras in bad weather conditions. A good proposal for car tracking using Kalman filtering was made by Li et al. [12]. The authors propose a new method relying on Kalman filtering to predict what they call the sheltered car moving position. However, the tracking efficiency still admits some improvement.

More recently, the particle filter has grown in popularity due to its flexibility and performance. Chan et al. [13] presented a method to detect and track the vehicle under various lighting conditions. The authors achieved this goal by generating a probability distribution of vehicles, using a particle filter framework, through the processes of initial sampling, propagation, observation, cue fusion and evaluation. The detection rate of their method under normal weather condition is 99.37%, the situation of the camera is fixed and they do not test the method under complicated weather condition (such as dusty, snowy, night, etc.).

Generalized particle filters provide a more powerful estimation for non-linear and non-Gaussian when compared to other estimators of non-linear systems for instance, the extended Kalman Filter. The main advantage of the GPF is that the particles do not depend on the linearization of the estimated non-linear method. The movements of vehicles aren’t linear and there aren’t dynamic equation sets that indicate their path in common. Generalized particle filters are able to estimate large non-linear dynamical systems sets, hence they are useful regardless of the cost of computation. GPF can be computed at a low cost.

Generalized Particle Filter (GPF) procedures are applied to track and identify small movement of vehicles, based on the generalized probabilistic distribution, and the likelihood of the estimation of each GPF being sampled from the distribution function was computed. Generalized particle filters are a general approach for particle filters using a sequential Monte Carlo algorithm. The generalized gamma distribution given in Eq 1 has been used for particle representation.

$$P_{x}\;(X\;|\alpha ,\beta ,\gamma )=\frac{\gamma \beta^{-\alpha \gamma }}{2\Gamma (\alpha )}\;{\left|X\right|}^{\alpha \gamma -1\;}\;\exp[-{\left(\frac{\left|x\right|}{\beta }\right)}^{\gamma }]$$ (1)

For tracking the vehicle, we stored the set of vehicle states as *x*_0_,*x*_1_,…,*x*_*t*−1_.*X*_*t*_ is the state of the position of a vehicle at the time t = 1, …, n, where n *is* the number of frames. The tracking of the vehicle consists of three phases:

**Prediction:** Tracking the vehicle, followed by observational measurements as *Y*_*t*_ where *y*_0_,*y*_1_,…,*y*_*t*−1_ is a set of measurements of the frame at time t. This was used to define *P* (*X*_*t*_|*Y*_0_ = *y*_0_,…,*Y*_*t*−1_ = *y*_*t*−1_).

**Data association:** we have used the formula *P* (*X*_*t*_|*Y*_0_ = *y*_0_,…,*Y*_*t*−1_ = *y*_*t*−1_) to determine some measurements obtained from frame t.

**Correction:** there are relevant measurements, so we computed the representation of *P*(*X*_*t*_|*Y*_0_ = *y*_0_,…,*Y*_*t*_ = *y*_*t*_). During vehicle tracking, we assume that *P*(*X*_0_) represents the probability of the first state in the position of the vehicle, with observation *Y*_0_, so we have;

$$P(X_{t}|Y_{0}=y_{0})=\frac{P(y_{0\;}|X_{0})\;P(X_{0})}{P(y_{0})}$$ (2)

We must initialize with a diffuse prior of a special form that is easily sampled

$$P(X_{t}|Y_{0}=y_{0\;},\ldots ,Y_{t-1})=\int P\;(X_{t\;},X_{t-1}|\;y_{0},\ldots ,y_{t-1\;})dX_{t-1\;}$$ (3)

The posterior density function *P*(*X*_*t*_|*Y*_0_ = *y*_0_,…,*Y*_*t*−1_ = *y*_*t*−1_) at each time t can be obtained recursively in two steps, namely prediction and update. The prediction step uses the probabilistic system transition model *P*(*X*_*t*_|*X*_*t*−1_) to predict the posterior distribution of *X*_*t*_ given all available observations *y*_0:*t*−1_ = {*y*_0_,*y*_1_,…,*y*_*t*−1_} up to time t − 1, and is recursively computed by

$$P(X_{t}\;|Y_{0}=y_{0\;},\ldots ,Y_{t-1}=y_{t-1})=\int P(X_{t}|\;X_{t-1})P(X_{t-1}\;|y_{0\;},\ldots ,y_{t-1})dX_{t-1}$$ (4)

*P*(*X*_*t*−1_|*y*_0_,…,*y*_*t*−1_) is the previous posterior at time t -1.

The correction involves obtaining the representation of *P*(*X*_*t*_|*y*_0_,…,*y*_*t*_) It is possible to write *P* (*X*_*t*_|*y*_*t*_) as;

$$P(X_{t\;}|y_{t})=\frac{P(y_{t\;}|X_{t})P(X_{t}|y_{0,\ldots ,}y_{t-1})}{\int P(y_{t}|X_{t})P(X_{t}|y_{0,\ldots ,}y_{t-1})dX_{t}}$$ (5)

*P*(*Yt* | *Xt*) represents the likelihood that will be computed by comparing predictions of the image with the actual image. As the likelihood must have many peaks, they must be monitored, so that the increase in the number of frames and particles can provide quality data for tracking. The generalized particle filter method is a sampling method for approximating the generalized distribution that makes use of its temporal structure and should handle multiple peaks and high-dimensional state vectors without difficulty. In order to compute the expected state of a vehicle given some information, we need to calculate the variance of the state, thus, the probability distribution is a device for accurately calculating expectation.

**Sampling representation:** The main function of the generalized particle filter method is sampling the representation of the probability distribution. Sampling representation of the likelihood from a collection of *x*_*t*_ | *i* = 1,…,*N*_*s*_ samples (particles) at time t and an associated collection of weights *wt*, where *x is* the numbers of samples for this state. These points are independent samples drawn from the probability distribution function *P*(*x*_*t*_|*x*_*t*−1_).The normalization of weights is given by

$$\sum_{t=1}^{Ns}w_{t}=1$$ (6)

The discrete weight is approximated to a posterior probability distribution function (pdf).

$$p\;(x\;(t)|y_{0:t})=\sum_{t=1}^{Ns}w_{t}\delta (x(t)-x_{t})$$ (7)

Here, δ(x) denotes the Dirac impulse function.

Where *N*_*s*_ (number of particles) must be large enough to converge on the true pdf. The samples *x*_*t*_ that are drawn from the importance distribution function are given by *q*(*x*_*t*_|*x*_0:*t*−1_,*y*_*t*_) so the weights are then updated (10) by combining these two Eqs (8 and 9).

The GPF recursively updates the particle location and the corresponding weights of the particles. The location and weight of each particle reflect the value of the posterior density in the region of the state space.

Measurement update for *x*_*t*_ | *i* = 1,…,*N*_*s*_, where ‘c’ is a normalization constant and *p*(*y*_*t*_|*x*_*t*_) is the likelihood function (8);

$$w_{t}=\frac{1}{c_{t}}\;w_{t}\;p(y_{t}|x_{t})$$ (8)

And compensate for the importance weight (9);

$$w_{t+1\;}=w_{t}\;\frac{p(x_{t+1\;}|x_{t})}{q(x_{t+1}|x_{t,}y_{t+1})}$$ (9)

Then, weight is updated according to the Eq (10);

$${\;w}_{t}\;\alpha \;\frac{p(y_{t}\;|x_{t})\;p\;(x_{t}\;|x_{t-1})}{q\;(x_{t\;}|x_{0:t-1,}y_{t})}$$ (10)

Using classical Monte Carlo integration, it is difficult to draw samples from the desired distribution. Importance sampling solutions are drawn samples from another proposal distribution and are weighted according to how they fit the original distribution. GPF has two main advantages over importance sampling. It can be used for an unlimited number of variables and the particles better cover the hypothesis space.

GPF is a sampling method that starts with a population of particles, each of which assigns a value to no variables, and has a weight of 1. Each step it can select a variable that has not been sampled and is not observed. For each particle, it samples the variable according to some proposal distribution.

Generalized particle filters can operate as importance samplers for this distribution because the importance sampling technique is a method of generating samples with the importance density as the prior density (11). Thus, the weight is updated according to Eq (12);

$$q(x_{t}\;|x_{0:t-1},y_{t})=p(x_{t}|x_{t-1})$$ (11)

$$w_{t\;}aw_{t-1}\;p(y_{t}|x_{t})$$ (12)

At any time t, the distribution is represented by the weighted set of samples. The probability of drawing each particle is given by its importance weight.

**Parameter estimation:** We used the maximum likelihood technique to estimate the parameters of the proposed distribution. In this application, the motion model which includes noise is defined by Eq 13.

$$\left\{ \begin{array}{c} x_{t+1\;}={Ax}_{t}+\;B_{u}\;u_{t\;}+\;B_{f}\;f_{t}\\ y_{t}=h(x_{t})+n_{t} \end{array}\right.$$ (13)

Where *x*_*t*_ is the state vector, *u*_*t*_ is measured in inputs, *f*_*t*_ is faults, *y*_*t*_ is measurements and *n*_*t*_ is the noise model. Although in different works a Gaussian distribution is preferred for the noise model, in this work we used a more realistic noise model to develop tracking. In our approach, the noise model is specified by two parts as shown below. One part of the noise model is given for density measurements, and a second part of the noise model is given for speed measurements.

$$p(n)=\sum_{n_{s}=0}^{\infty }\frac{\lambda_{1}^{\left(n+\;n_{s}\right)}\;e^{{-\lambda }_{1}}}{(n+n_{s})!}\times \frac{\lambda_{1}^{\left(n+\;n_{s}\right)}\;e^{-\lambda_{1}}}{n_{s}}\;$$ (14)

Here, we assumed that the motion of the particle as a constant velocity assumption. We used a constant speed noise model in this paper, since the relative speed during a short time interval can be regarded as a constant. Thus, we add the speed component to the noise model. Also, the scalar noise is in the nature and all three color RGB have the same average noise magnitude with constant noise. Eq 14 is an exponential function and it allows for the compact expression.

Light consists of particles called photons, each of which has an energy hv, where h (= 6.626 × 10^−34 *Js*^) is Planck’s constant and v is the frequency. As the frequency is related to the wavelength by *s* = *vλ*. s is the speed of light (= 2.998 × 10^8^ *ms*^−1^). The noise power spectrum is constant. This noise power is the equivalent to the power spectral density function.

In this work, based on noise area consideration, we assumed that the noise speed *n*_*s*_ is Gaussian and $\lambda_{1}+\;\lambda_{2}=2,\lambda_{1}=\frac{4}{3},\lambda_{2}=\frac{2}{3}$. Also, we assumed independent distributions for *f*_*t*_, *n*_*t*_ and x, with known probability densities that were not necessarily Gaussian.

### 4.1 Decision making

After these stages, we used Eq 15 to make a decision about the vehicles and count them. For this purpose, we compared the estimated locations $P_{t-1}^{estimated\;}$, with detected blobs of vehicles, $P_{t}^{detected}\;$ in the current frame.

$$Final\_result=\left\{ \begin{array}{l} \;1\;ǁ\;P_{t}^{detected}-\;P_{t-1}^{estimated\;}ǁ\;<\;\delta \\ \;0\;\mathrm{else} \end{array}\right.$$ (15)

Where, ǁ.ǁ is norm 2 criterion used as the distance measurement function and δ was a small constant (3 in this work).

## 5. Experimental results

**Fig 4:**
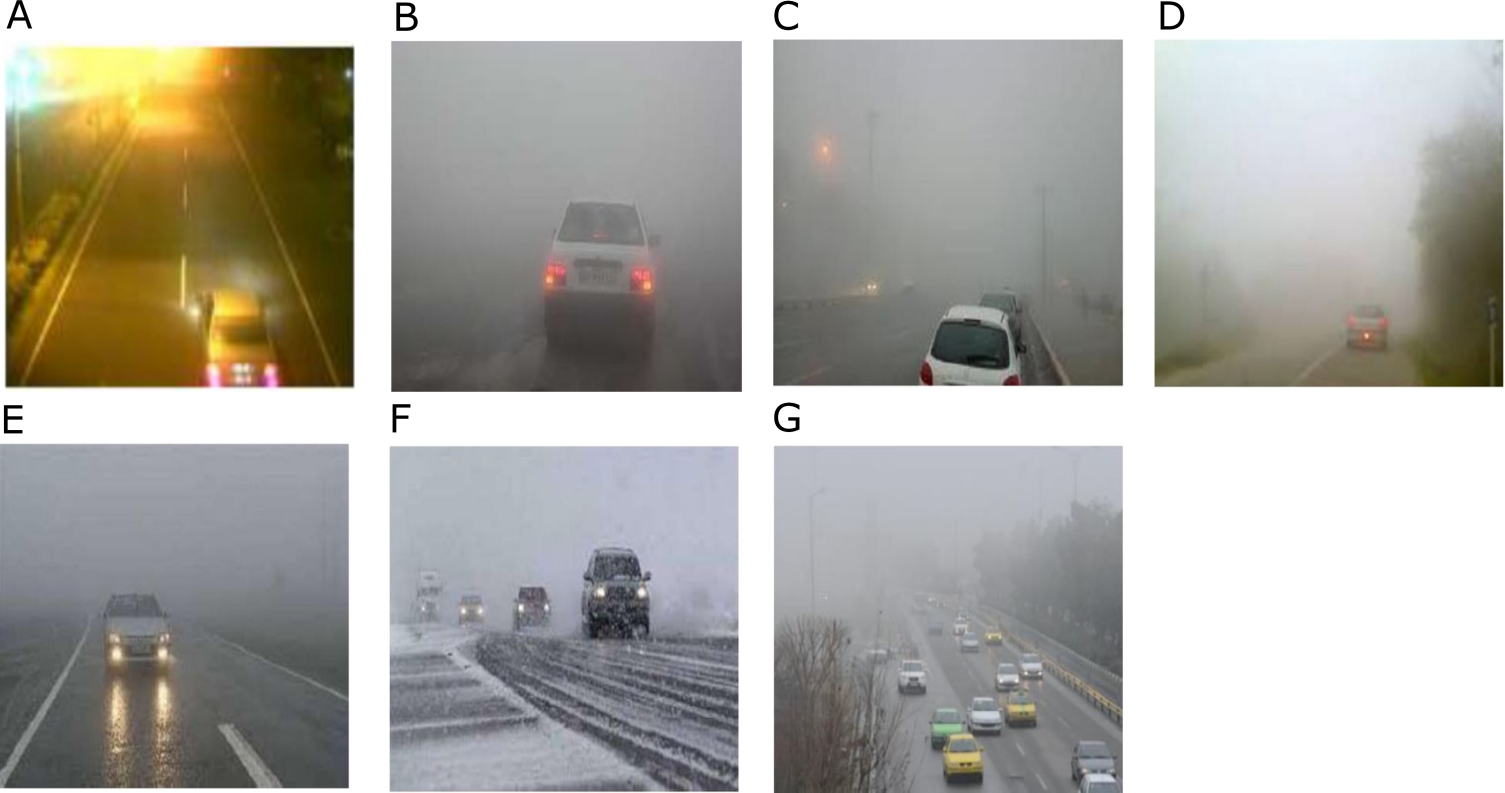
**Samples of Datasets (a-g).** (a)Foggy weather, Bad lighting condition, 364*470 resolution, 187 frames. (b) Foggy and snowy weather, 364*470 resolution, 190 frames. (c) Foggy weather and Occlusion, 364*470 resolution, 197 frames. (d) Foggy weather and vibrating camera, 364*470 resolution, 186 frames. (e) Foggy, rainy weather and vibrating camera, Bad lighting condition, 364*470 resolution, 201 frames. (f) snowy weather and vibrating camera, 364*470 resolution, 185 frames. (g) Foggy weather and vibrating camera, Occlusion, 364*470 resolution, 186 frames.

The proposed method was implemented in Matlab framework. Our method could process around 8 frames per second on a dual core processor at 2.4 GHz. We used video data sets from highways which had unfavorable conditions such as dusty weather with vibrated camera. For fair evaluation of our proposed method, we used test videos in different conditions, Fig 4. These conditions were bright night, dusty and snowy weather with vibrated camera. To evaluate the performance of the system, we used to test video data collected under various weather and lighting conditions, over a period of more than seven days. The data set made contains video data collected at different area in Iran and under different conditions, such as vibrating camera, rainy, snowy, dusty or foggy weather and collision. We collected more than 50 videos, with up to 5 minutes length each one. These videos contain up to 2100 frames; the initial frames, nearly 20 percent, do not contain any vehicle. Fig 4 shows those videos corresponding to the unfavorable weather conditions.

**Fig 5:**
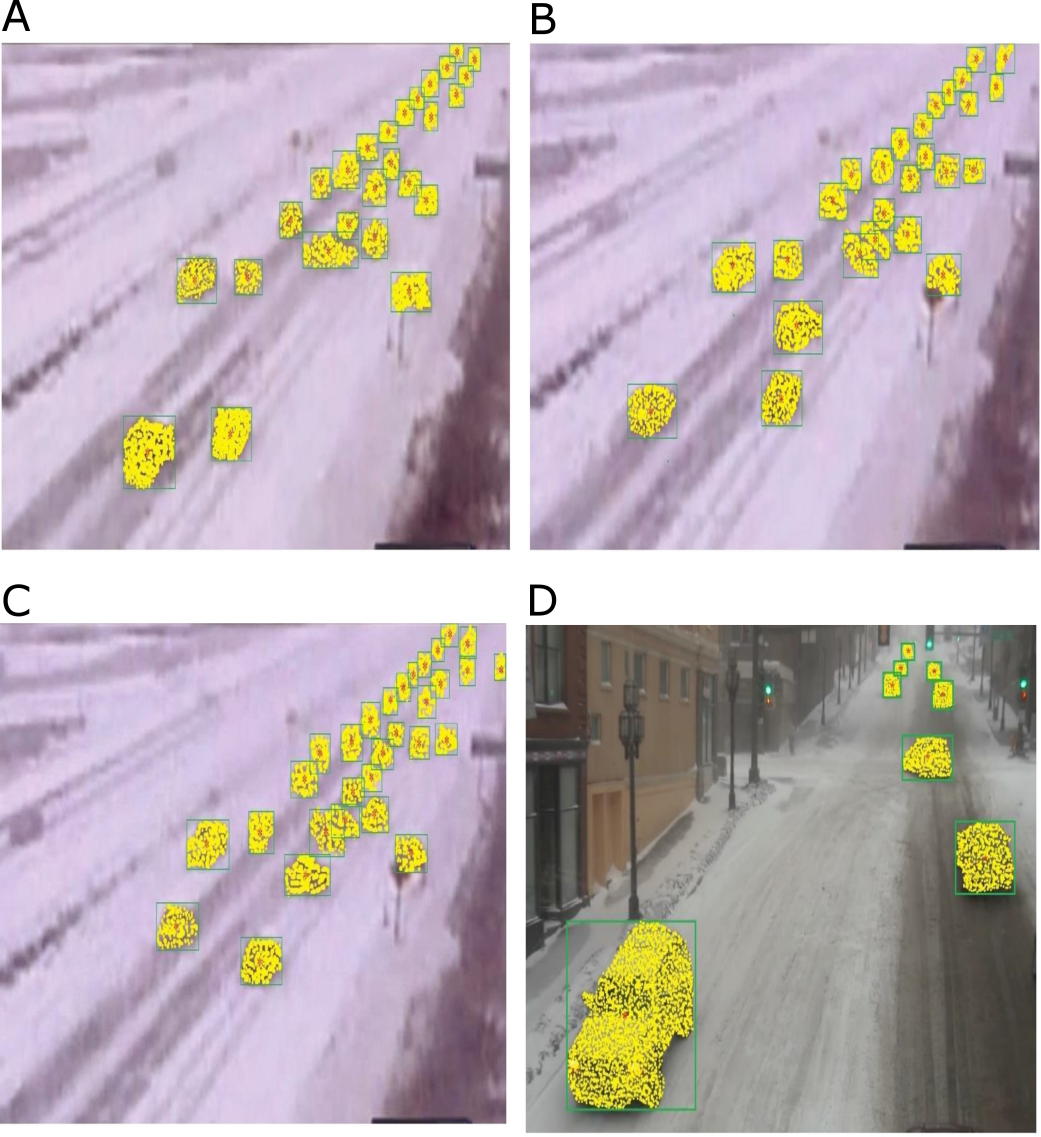
Showing the particles drawn on the vehicles using (GPF) a β-distribution function. Including snowy weather (snow in the air and on the ground) with the camera vibrating due to strong wind (which make the picture unclear) (a-d). (a) snowy weather with vibrating camera, Frame 408 (26 vehicles tracked). (b) snowy weather with vibrating camera, Frame 453 (26 vehicles tracked). (c) snowy weather with vibrating camera, Frame 470 (30 vehicles tracked). (d) foggy weather after snow, Frame 63 (8 vehicles tracked).

We applied the new proposed method with the generalized probability distributions and different parameters, leading to different results. Fig 5 shows vehicle tracking using the β-distribution function, with 2000 particles on some frames including snowy weather (snow in the air and on the ground) with the camera vibrating due to strong wind (which make the picture unclear).

**Fig 6:**
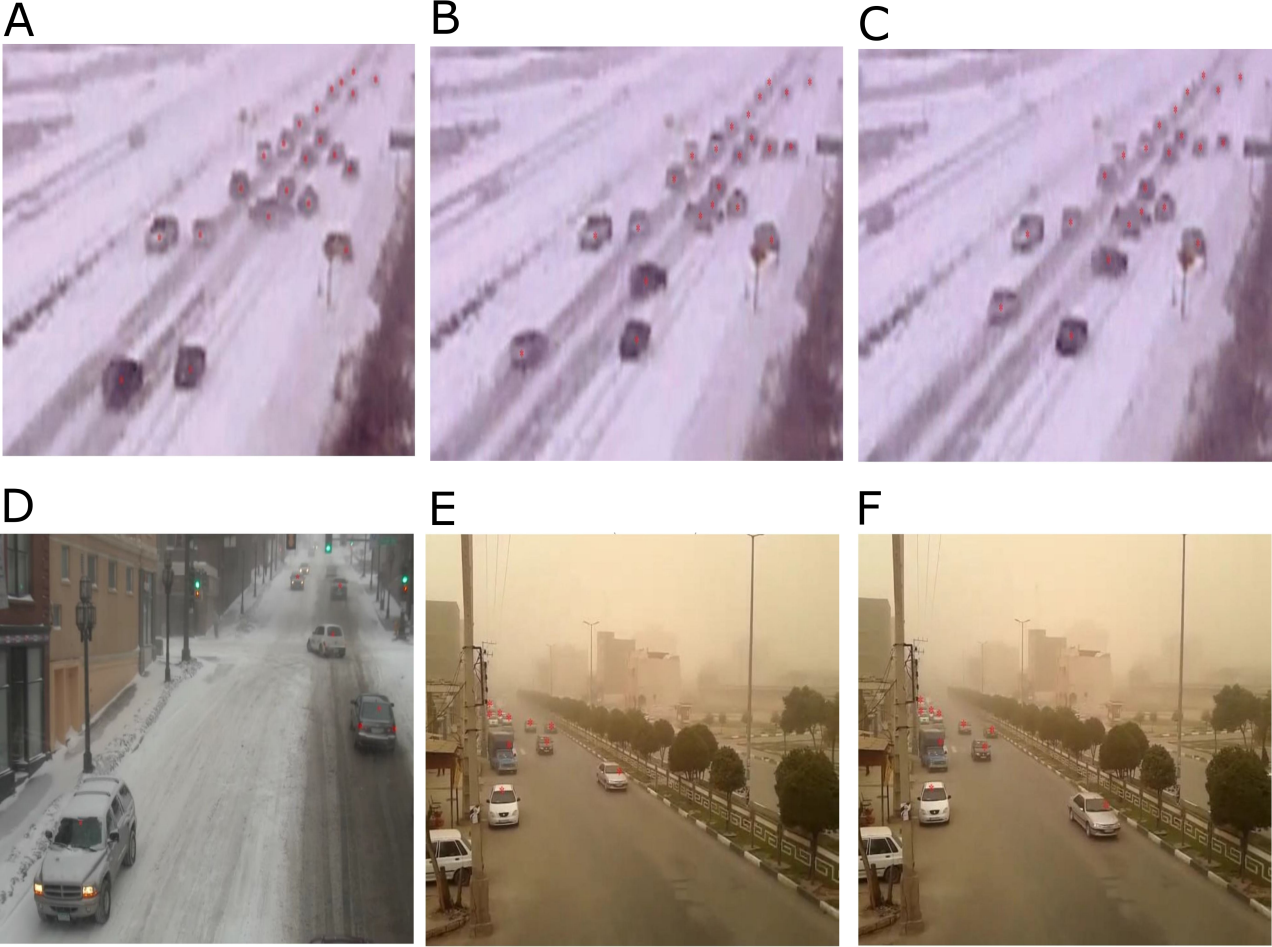
Examples of simultaneous tracking of vehicles, using particle filter. (a, b, c,) Including snowy weather (snow in the air and on the ground) with vibrated camera conditions due to strong wind, which make the picture unclear. (a) Frame 408 (22 vehicles tracked), (b) Frame 453 (23 vehicles tracked), (c) Frame 470 (23 vehicles tracked). (d) Including foggy weather after snow (snow on the ground), Frame 63 (5 vehicles tracked). (e, f) Including dusty weather with vibrated camera conditions due to strong wind, which make the picture unclear. (e) Frame 2099 (10 vehicles tracked). (f) Frame 20114 (10 vehicles tracked).

Fig 6 Shows simultaneous tracking of vehicles, using particle filter [10]. In the Fig 6A we had 22 vehicles tracked, but using generalized particle filter increased the tracking rate to 26 vehicles in the same frame Fig 5A. In the Fig 6C we had 23 vehicles tracked with the particle filter, but using GPF the tracking rate was increased to 30 vehicles in the same frame Fig 5C. Also, our GPF increased the tracking rate in the dusty weather with vibrated camera conditions. Fig 6E and 6F are randomly selected from our tracking result with the particle filter.

**Fig 7:**
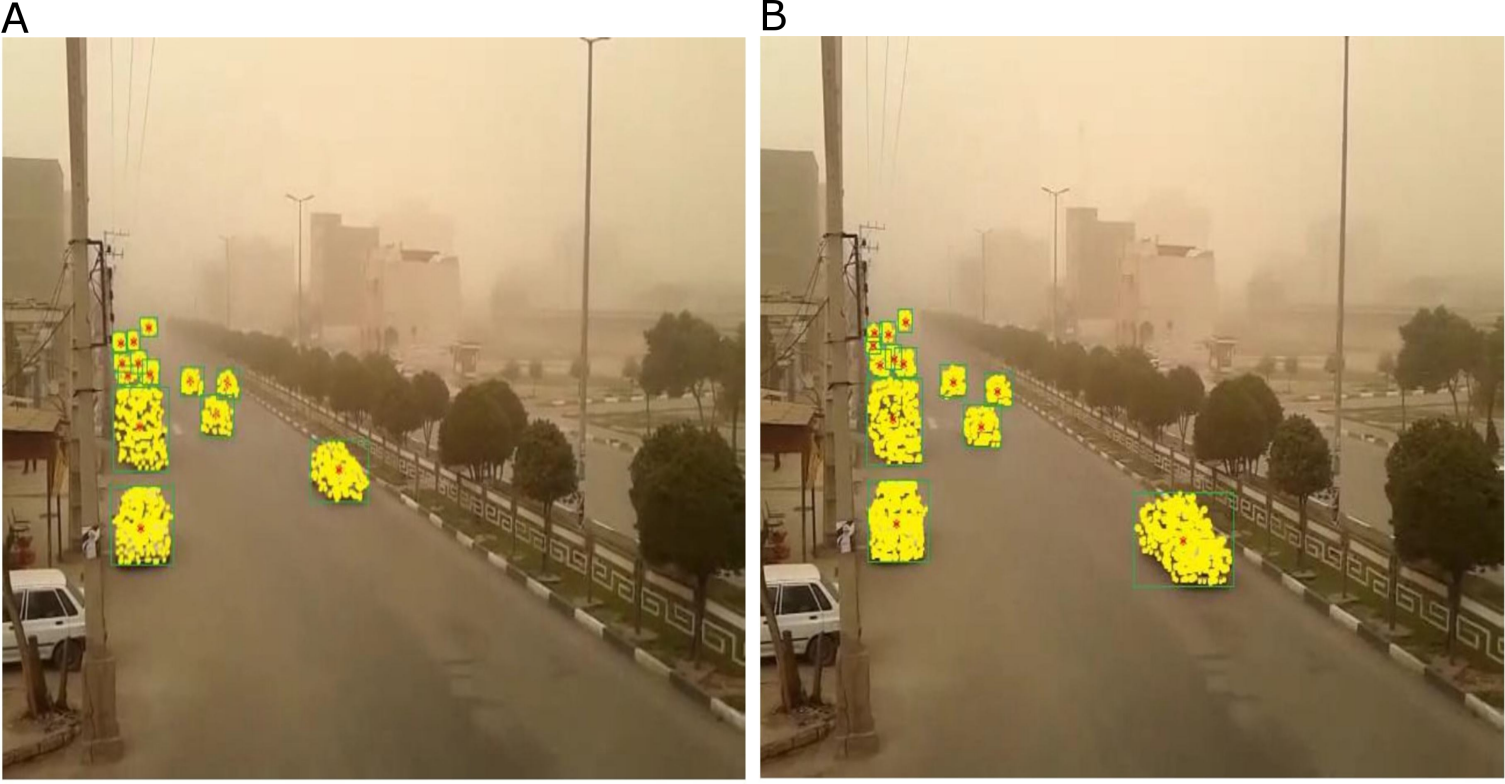
Showing the particles drawn on the vehicles using (GPF) a β-distribution function. Including dusty weather with vibrated camera conditions due to strong wind (which make the picture unclear) (a-b). (a) Frame 2099 (13 vehicles tracked). (b) Frame 20114 (13 vehicles tracked).

Our method worked suitably in ALL conditions. Our simulation results confirmed the better performance of our method. Fig 7 shows our new method with generalized particle filter in some frames (dusty weather with vibrated camera condition). Fig 7A and 7B are tracking results with the generalized particle filter in the same frames.

Result indicates that we have better detection and tracking rate even far from the vibrated camera in snowy and dusty weather conditions according to our previously published method tracking system with particle filter [10].

Even though the videos gather different field of views, our system is able to monitor an area of approximately 80×12 meters. With this field of view, we are able to increase detection and tracking rate simultaneously the set of vehicles present in the monitored area, which may rise to 30 as maximum, more or less.

In our testing scenario, we used a trajectory mechanism to evaluate our method. For this purpose, we considered several situations of vehicles in our trajectory and adapted our estimations to the detected ones. Our proposed method used estimation data to refine its detection results for more efficient counting. In other words, when we faced frames with occlusion between vehicles or the frames had undesirable blobs due to dusty weather with vibrated camera, our estimation results which tracked the previous frames helped us to make a decision about vehicles in the current frame.

The details and comparison of the detection results of our proposed method with other methods in different conditions are shown in Table 1. In this table, ‘Correct’ means the number of vehicles detected correctly and ‘Missed’ means the number of missed vehicles. We randomly selected 50 frames for each weather condition. Note that the number of vehicles were counted manually (Ground Truth). Detection Rate (DR) or Recall DR = TP/ (TP+ FN)* 100.

**Table 1:** Comparison of detection rate of our proposed method (GPF) with other previously published methods.

Methods	Ground truth	Correct				Missed				Detection Rate %			
		PF[10]	GPF	[7]	[13]	PF[10]	GPF	[7]	[13]	PF[10]	GPF	[7]	[13]
Normal	455	450	453	442	452	5	2	13	3	98.9	99.83	97	99.34
Dusty	806	654	780	591	648	152	26	215	158	81.1	94.78	73.3	80.39
Vibrated Camera	307	245	299	239	240	62	8	68	67	79.8	97.56	77.8	78.1
Snowy	283	236	270	231	235	47	13	52	48	83.4	96.97	81.6	83.03

Based on the results of Table 1, the detection rate of the new proposed method increased. For normal weather condition is up to 99.83%. However, our performance in dusty weather has 94.78% of success rate detection although our strategy is new in unfavorable conditions, we used a detection and counting method based on the Kalman filter [14] to compare the performance of our proposed method. This algorithm is well known and is referenced by many researchers in similar works [14, 15]. The detection rates of this method in similar condition are mentioned in Table 2.

**Table 2:** Detection rate of Kalman based method.

Category	Correct	Missed	Detection rate%
Normal	452	3	99.3
Dusty	589	217	73
Vibrated camera	187	120	61
Snowy	195	88	69

### 5.1 Speed estimation

The distance (d) traveled by the vehicle between two successive frames is calculated by centroid of blobs. Distance between 2 centroid P (xi,yi, zi) and Q(xj,yj, zj) in world space.

$${dist}_{ij}=\sqrt{{\left(x_{i\;}-x_{j}\right)}^{2}}+\;{\left(y_{i\;}-\;y_{j}\right)}^{2}+\;{\left(z_{i}-z_{j}\right)}^{2}$$ (16)

n = total number of frames

In order to calculate the speed, we obtain the time of motion using the following formula. Time of motion in seconds is calculated as:

$$t=\frac{n}{fps}$$ (17)

Where fps = frame rate (number of frames per second) Estimated velocity of the vehicle in meters per second is calculated as:

$$Vel\;\left(\frac{cm}{s}\right)=\frac{d(cm)}{t(s)}$$ (18)

The experimental results for speed estimation of vehicles are shown in Table 3 and the error is calculated. The error is calculated by comparing the detected speed and the real speed which is obtained from traffic center in Iran. (There is no vehicle with a speed of more than 100 (km/h), because of bad weather conditions).

**Table 3:** Experimental result for speed estimation in different weather conditions with vibrated camera.

Vehicle	Frame rate	Real Speed Average (km/h)	Detected Speed Average(km/h)	Error (km/h)
Dusty weather 1	30	27	27.5	0.50
2	30	22.4	23.4	1
3	30	33.5	33.9	0.4
4	30	36.1	37.9	1.8
5	30	26.8	27.3	0.5
6	30	85.2	85.7	0.5
7	30	77.9	78.2	0.3
Snowy weather 1	30	22.5	22.9	0.4
2	30	31.9	32.6	0.7
3	30	19.5	20.7	1.2
4	30	24	24.3	0.3
5	30	23.7	23.9	0.2
6	30	73.5	73.9	0.4
7	30	65.8	66.1	0.3
Night 1	30	50.3	51.7	0.4
2	30	45	46.8	1.8
3	30	45.7	47.8	2.1
4	30	52	52.1	0.1
5	30	54.1	54.9	0.8
6	30	96.5	96.9	0.4
7	30	90.8	91.5	0.7

As shown in Table 3, the error occurs because of the vibrating of video which induces change in filter output.

## 6. Conclusion and future work

In this paper, we proposed a new method to improve the detection rate and counting of vehicles in dusty and snowy weather conditions with a vibrating camera. Our method uses an improved background subtraction algorithm to detect vehicles. Our innovation in this section includes a series of processes combined with a background subtraction algorithm to remove virtual blobs and decrease the effect of dusty weather conditions with a vibrating camera. Additionally, we used a tracking step to achieve more data for the verification of vehicles in each frame based on their information in previous frames.

This idea was implemented by a generalized particle filter and improved our detection and counting procedure. Our results showed that our proposed method was robust in complex conditions such as bright nights and snowy and dusty weather conditions with a vibrated camera (due to the wide range of traffic conditions and varying speeds in different lanes). Our results are comparable to other works, our previously published method with a particle filter and those that use the Kalman filter in normal conditions, but show much better results under extreme conditions, such as dusty or snowy weather, with a vibrating camera. The accurate tracking information provided allows our system to face the next step, which is to measure the average speed of the vehicles in the monitored field of view. We calculated the speed of vehicles in complicated conditions with less errors.

For future work, it would be convenient to include some information that would allow the quantification of the blurring level of the images, according to the amount of snow, dust or fog captured by the camera. To achieve that, we have plans to incorporate an opacimeter in the monitored areas, so that we can characterize the level of noise present in the images and, with that, provide estimated operational conditions for our proposal.

## Supporting information

S1 File Some examples of tested Dataset. Recorded in Spain and Iran.
(DOCX)
