# Correction: Improving vehicle tracking rate and speed estimation in dusty and snowy weather conditions with a vibrating camera

**DOI:** 10.1371/journal.pone.0197446

**Published:** 2018-05-10

**Authors:** Nastaran Yaghoobi Ershadi, José Manuel Menéndez, David Jiménez

Dr. José Manuel Menéndez should be included in the author byline. He should be listed as the second author, and his affiliation is 1: E.T.S. de Ingenieros de Telecomunicación, Universidad Politécnica de Madrid, Madrid, Spain. The contributions of this author are as follows: Methodology, Writing–original draft, Writing–review & editing, and supervision.

Dr. David Jiménez should be included in the author byline. He should be listed as the third author, and his affiliation is 1: E.T.S. de Ingenieros de Telecomunicación, Universidad Politécnica de Madrid, Madrid, Spain. The contributions of this author are as follows: Conceptualization, Validation, and Writing–review & editing.

The correct citation is: Yaghoobi Ershadi N, Menéndez JM, Jiménez D (2017) Improving vehicle tracking rate and speed estimation in dusty and snowy weather conditions with a vibrating camera. PLoS ONE 12(12): e0189145. https://doi.org/10.1371/journal.pone.0189145
