# Supplementary material for: Improving vehicle tracking rate and speed estimation in dusty and snowy weather conditions with a vibrating camera
Source: PLoS One. 2017 Dec 20;12(12):e0189145. doi: 10.1371/journal.pone.0189145 (PMC5738070; doi:10.1371/journal.pone.0189145)

Please Note that, because of the bad weather conditions such as dusty, snowy, rainy, low light and vibrating of camera due to strong wind, the figures are unclear.


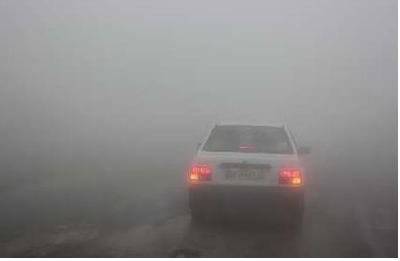


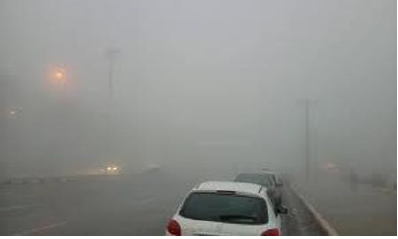


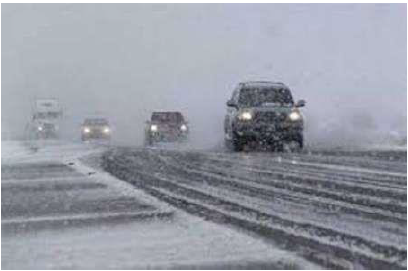


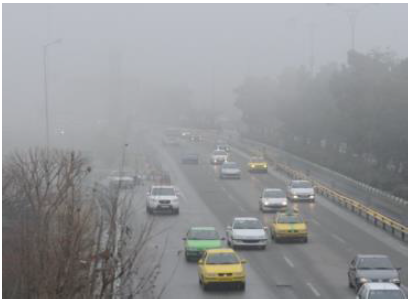


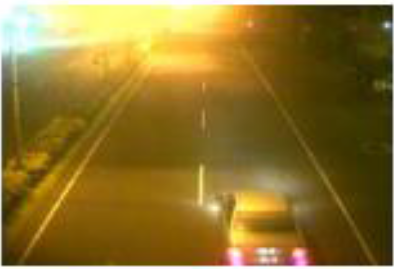


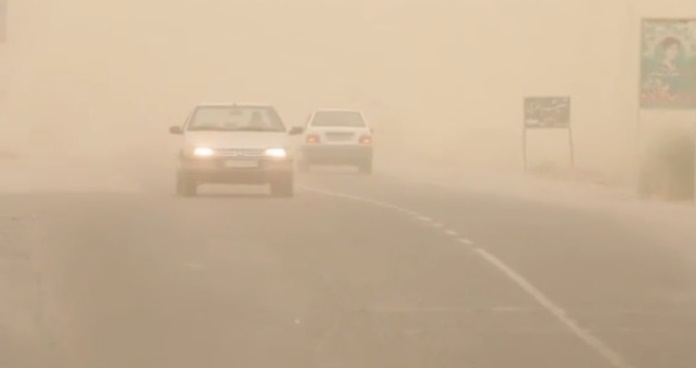


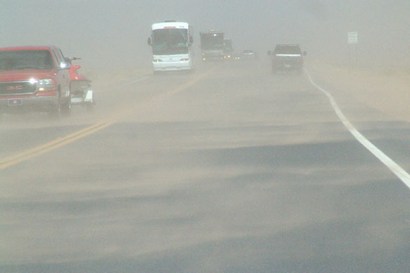


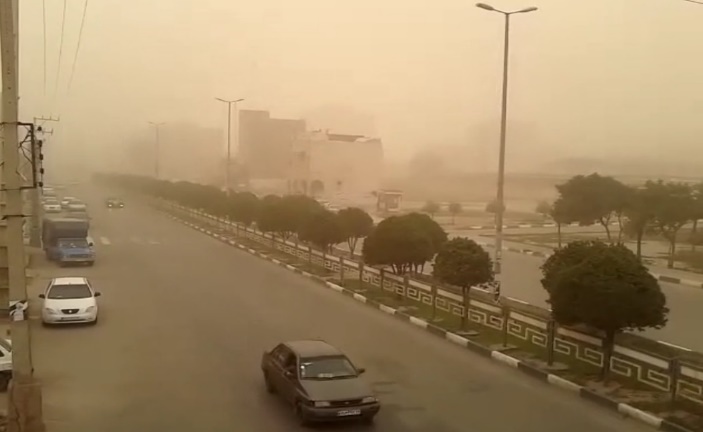


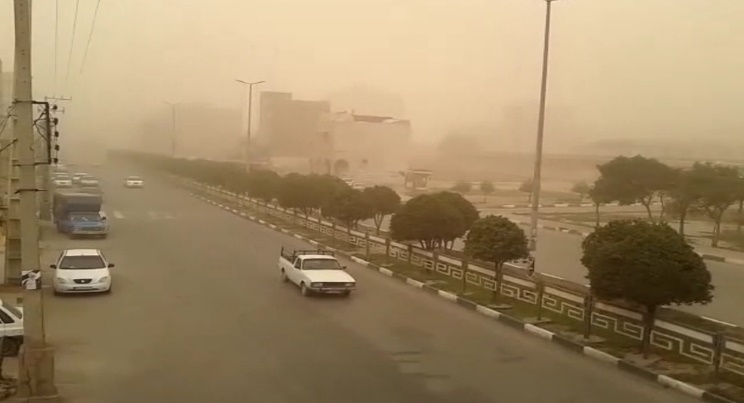


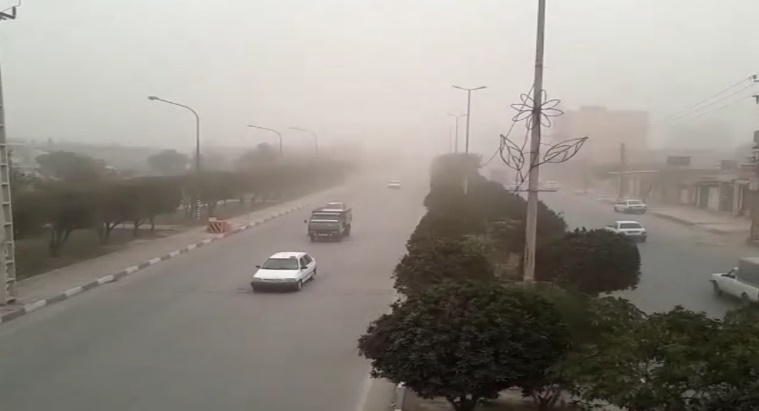


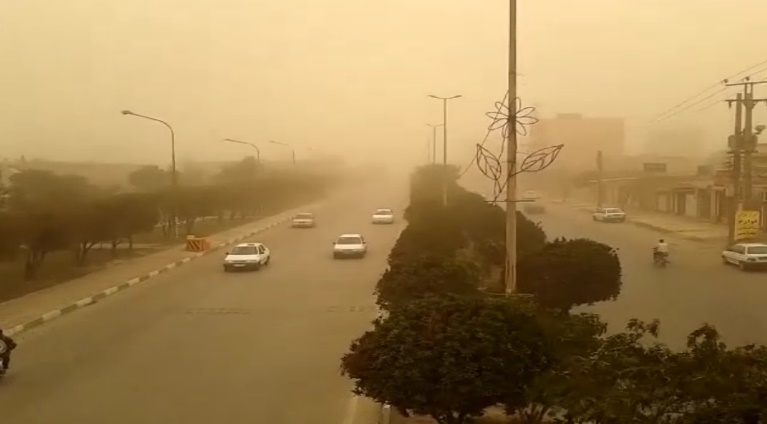


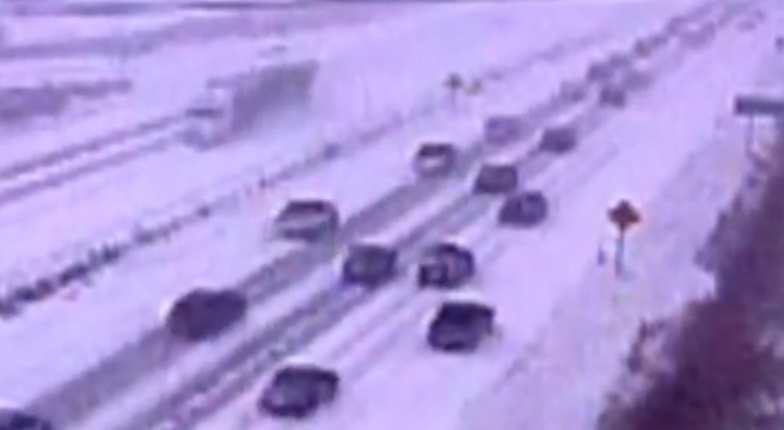


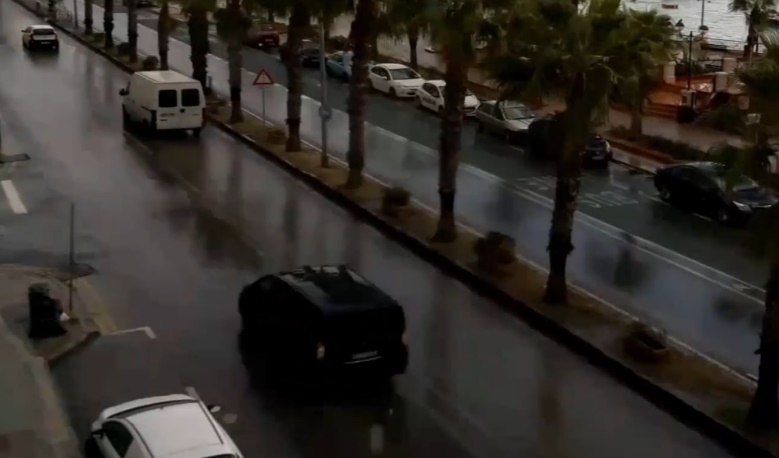

Supplement: S1 File — Recorded in Spain and Iran. (DOCX) [file pone.0189145.s001.docx]
